# Post-traumatic Pseudoaneurysm of the Internal Carotid Artery with Intractable Epistaxis: A Case Report

**DOI:** 10.31729/jnma.5860

**Published:** 2022-02-28

**Authors:** Subash Phuyal, Anisha Pandey, Suresh Bishokarma, Ritesh Lamsal, Gopal Sedain

**Affiliations:** 1 Department of Neuroimaging and Interventional Neuroradioiogy, Upendra Devkota Memorial National Institute of Neurological and Allied Sciences, Kathmandu, Nepal; 2 Department of Critical Care Medicine, Grande International Hospital, Kathmandu, Nepal; 3 Department of Neurosurgery, Upendra Devkota Memorial National Institute of Neurological and Allied Sciences, Kathmandu, Nepal; 4 Department of Anaesthesiology, Tribhuvan University Teaching Hospital, Kathmandu, Nepal; 5 Department of Neurosurgery, Tribhuvan University Teaching Hospital, Kathmandu, Nepal

**Keywords:** *aneurysm*, *epistaxis*, *internal carotid artery*, *pseudoaneurysm*

## Abstract

Epistaxis is a common otorhinolaryngology emergency. There are several treatment modalities for epistaxis, but bleeding from the internal carotid artery necessitates a particular treatment technique. We report a case of a 22-years old man who presented to us recurrent episodes of epistaxis and blurry vision in the right eye for one month. The patient had undergone maxillo-facial surgery following a road traffic accident one year back. Bleeding episodes were occasionally severe with blood loss of up to 800 to 1000ml. These episodes were managed conservatively with posterior nasal packing and frequent blood transfusions. A computed tomography-angiography revealed a pseudoaneurysm arising from the cavernous segment of the right internal carotid artery which was managed successfully by embolization of the aneurysm sac with coils. Despite the rarity of internal carotid artery pseudoaneurysm in individuals with a history of trauma, doctors must be aware of the possibility. Timely identification and treatment of a pseudoaneurysm can save a person's life.

## INTRODUCTION

Epistaxis is a common emergency in otorhinolaryngology. It is estimated that 7-60% of the population experiences epistaxis of varying severity at some point in their lives.^1^ Epistaxis may be caused by dryness of the nasal mucosa, trauma, hypertension, blood dyscrasias, and anticoagulant medications. Epistaxis is usually managed conservatively or with nasal packing. However, bleeding can sometimes arise from the Internal Carotid Artery (ICA) and requires a different management strategy. Although ICA pseudoaneurysm is rare, clinicians must be aware of the possibility in patients with a history of trauma. Timely diagnosis and prompt treatment of the pseudoaneurysm can be life-saving.

## CASE REPORT

A 22-years old patient presented to us after massive epistaxis that stopped spontaneously after about 400ml of blood loss. He had a recent history of recurrent episodes of nasal bleeding for one month. This was associated with blurring of right eye vision for the same duration. The patient had sustained facial trauma one year back and had undergone open reduction and internal fixation for Le Fort I and II fractures one year back. The patient reported that epistaxis was occasionally severe and would usually stop only after nasal packing. He was admitted to the special care unit and relevant laboratory investigations were requested. The hematocrit and haemoglobin levels were 24.1% and 7.9g/dl, respectively; the rest of the hematologic, biochemical, and coagulation parameters were normal.

**Figure 1:**
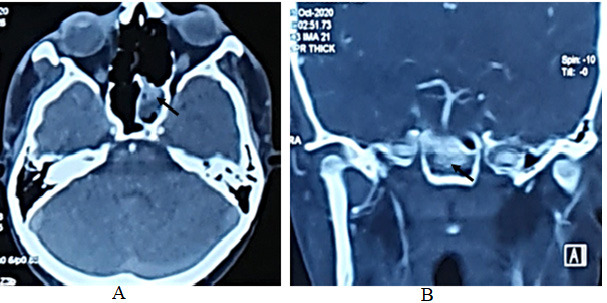
A) CT axial view shows blood and contrast within the sphenoid sinus (arrow), B) CT coronal view shows blood and contrast within the sphenoid sinus (arrow).

A contrast-enhanced computed tomography (CT) scan of the paranasal region was performed, which revealed blood within the sphenoid sinus with fixation plates for fracture of the right maxillary bone. CT angiography images showed an outpouching that protruded into the right sphenoid sinus arising from the medial aspect of the intracavernous portion of the right ICA. The outpouching measured approximately 4x4mm (Figure 1A, 1B), suggesting an aneurysm of the right ICA.

**Figure 2:**
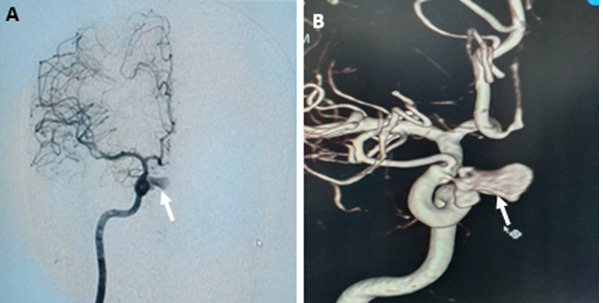

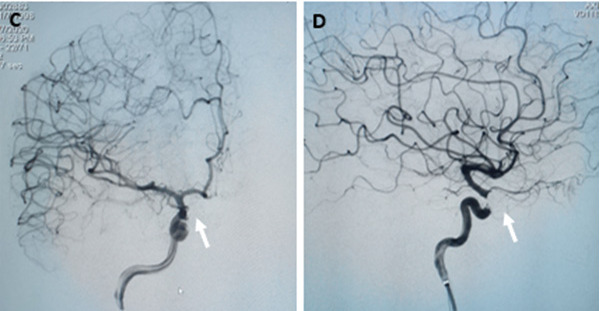
A) 2D right internal carotid artery angiogram shows an aneurysm in the intra-cavernous segment (arrow); B) 3D image shows the pseudoaneurysm near the origin of the right ophthalmic artery (arrow); C, D) 2D anteroposterior and lateral angiograms show complete obliteration of the pseudoaneurysm sac (arrows).

The patient was shifted to the cath lab to secure the aneurysm. After right transfemoral catheterization, a digital subtraction angiogram (DSA) of the cerebral vessels was obtained, which showed a pseudoaneurysm arising from the medial wall of the cavernous segment of the right ICA (Figure 2A, 2B). Before endovascular embolization, the presence of cross-flow of blood from the contralateral ICA and the vertebral arteries was confirmed. The pseudoaneurysm was treated with endovascular coils placed into the sac, preserving the parent artery. The post-procedure control angiogram showed complete occlusion of the pseudoaneurysm (Figure 2C, 2D). The patient remained stable for two days and was then discharged from the hospital. The patient was followed-up after six weeks and was free of all previous symptoms.

## DISCUSSION

Aneurysms of the cavernous segment of the ICA are rare and comprise only 3-5% of all intracranial aneurysms.^2^ The majority of these cases occur secondary to trauma. The hallmark of post-traumatic pseudoaneurysm is delayed, progressively severe, and sometimes fatal epistaxis. The average time from trauma to the rupture of pseudoaneurysm is around three weeks.^3^ Mortality rates of up to 50% have been reported in ruptured ICA pseudoaneurysms.^4,5^

Pseudoaneurysms commonly develop after tangential blunt or penetrating injury in an arterial wall through with blood continues to flow.^3^ The trauma can lead to an intimal tear resulting in decreased blood flow and hence, intraluminal thrombus formation.^6^ Patients with pseudoaneurysms of the cranial ICA may also be at an increased risk for developing distal thromboembolism, and occasionally, intracranial bleeding.^7^ Although small pseudoaneurysms of the ICA can be asymptomatic, they can sometimes present with massive epistaxis. As the cavernous part of the ICA indents the lateral wall of the sphenoid sinus, nasal bleeding can be a manifestation of a cavernous ICA aneurysm.^8^ Partial or complete ipsilateral monocular visual loss is also common. The vision loss can be caused by either compression or laceration of the optic nerve, or thrombosis of the ophthalmic artery. The oculomotor nerve and other divisions of the trigeminal nerve are seldom involved. The combination of head injury, monocular blindness and nasal bleeding constitutes the Maurer's triad.^9^

The DSA is the gold standard for the diagnosis of ICA aneurysms. A 3D CT-angiography is a less invasive alternative to the DSA. The definitive treatment of ICA pseudoaneurysms may be either open surgical or endovascular. Open neurosurgical options include direct clipping, wrapping, trapping and carotid artery ligation.^10^ However, of late, endovascular techniques have shown promising results.^11,12^ Among endovascular techniques, detachable balloons, and coils have been successfully used to treat these aneurysms.

Whenever performing endovascular obliteration of aneurysms, it is useful to perform a test occlusion of the ICA using a non-detachable balloon to determine the adequacy of collateral blood flow to the brain. Furthermore, the use of a detachable balloon allows not just the proximal occlusion of the ICA, but also the occlusion of the segment of the ICA distal to the aneurysm. In this manner, collateral blood flow to the aneurysm can be prevented and the likelihood of a rebleed and delayed embolic strokes are reduced.

Even though the pseudoaneurysm of the ICA is a rare entity, it must be considered among the differential diagnoses of intractable epistaxis, especially if there is a prior history of maxillofacial trauma. The mortality rate in ruptured ICA pseudoaneurysm is high; early diagnosis and treatment can be life-saving. The development of various endovascular techniques has simplified the definitive treatment of these complex aneurysms in recent times.
